# Supplementary material for: Screening and dynamic change study of microbial and metabolite markers for calf diarrhea based on multi-omics and machine learning
Source: mSystems. 2026 Apr 6;11(5):e00005-26. doi: 10.1128/msystems.00005-26 (PMC13185640; doi:10.1128/msystems.00005-26)
Supplement: Supplemental material — Fig. S1 to S5; Tables S1 to S6. [file msystems.00005-26-s0001.docx]

Supplementary Figures and legends


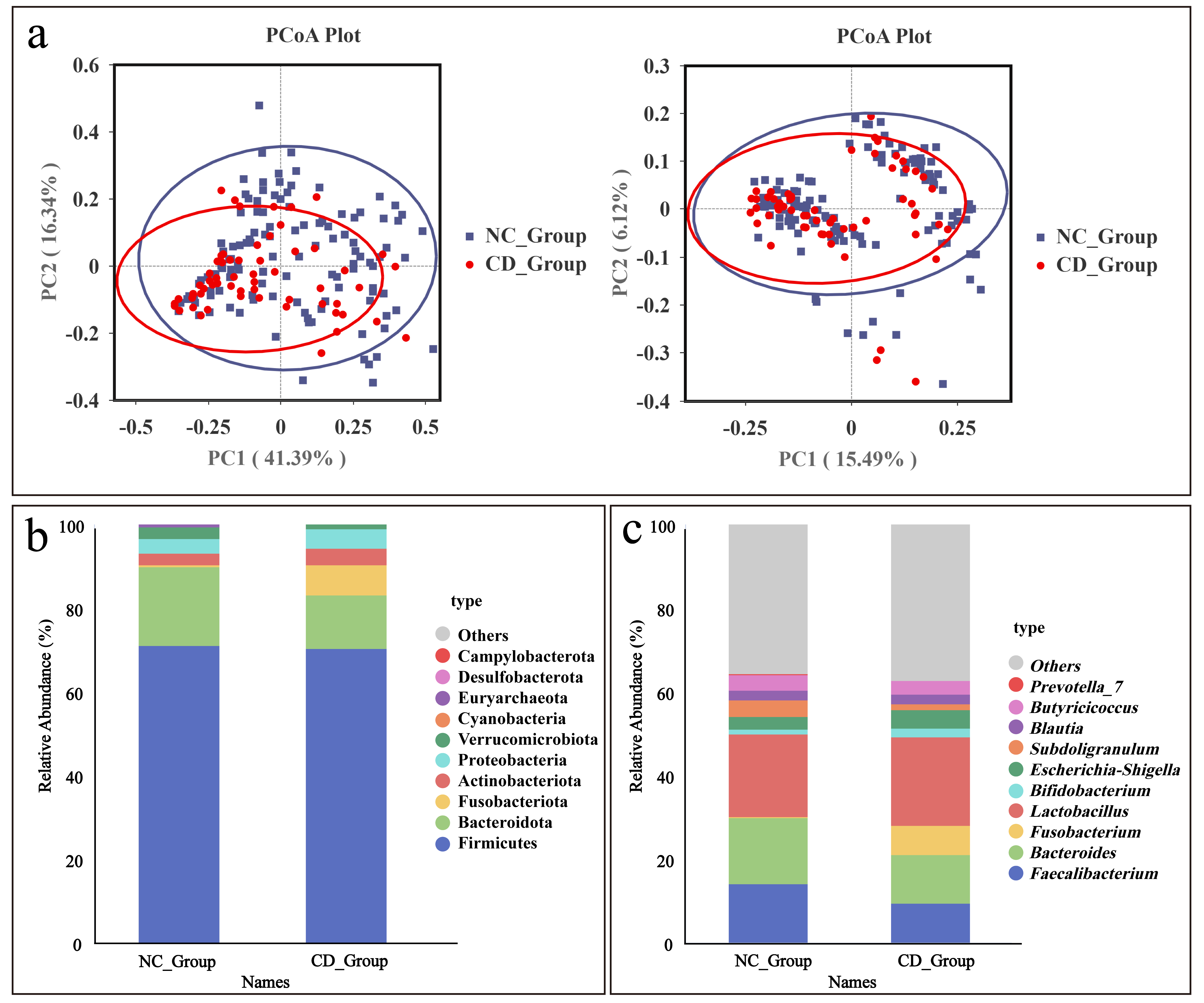


Fig S1. Fecal microbiota analysis in normal and diarrheal conditions. (a) β diversity was visualized by PCoA, using Weighted_Unifrac (left) and Unweighted_Unifrac (right). (b) Relative abundance of bacterial phyla. (c) Relative abundance of bacterial genera.





Fig S2. Differential metabolite dynamic changes. (a) Dynamics of differential metabolites in the negative ion mode during the occurrence of diarrhea over 10 days. (b~e) Dynamics of differential metabolites in the positive ion mode during the occurrence of diarrhea over 15 days.





Fig S3. Differential metabolite dynamic changes. (a~d) Dynamics of differential metabolites in the negative ion mode during the occurrence of diarrhea over 15 days.


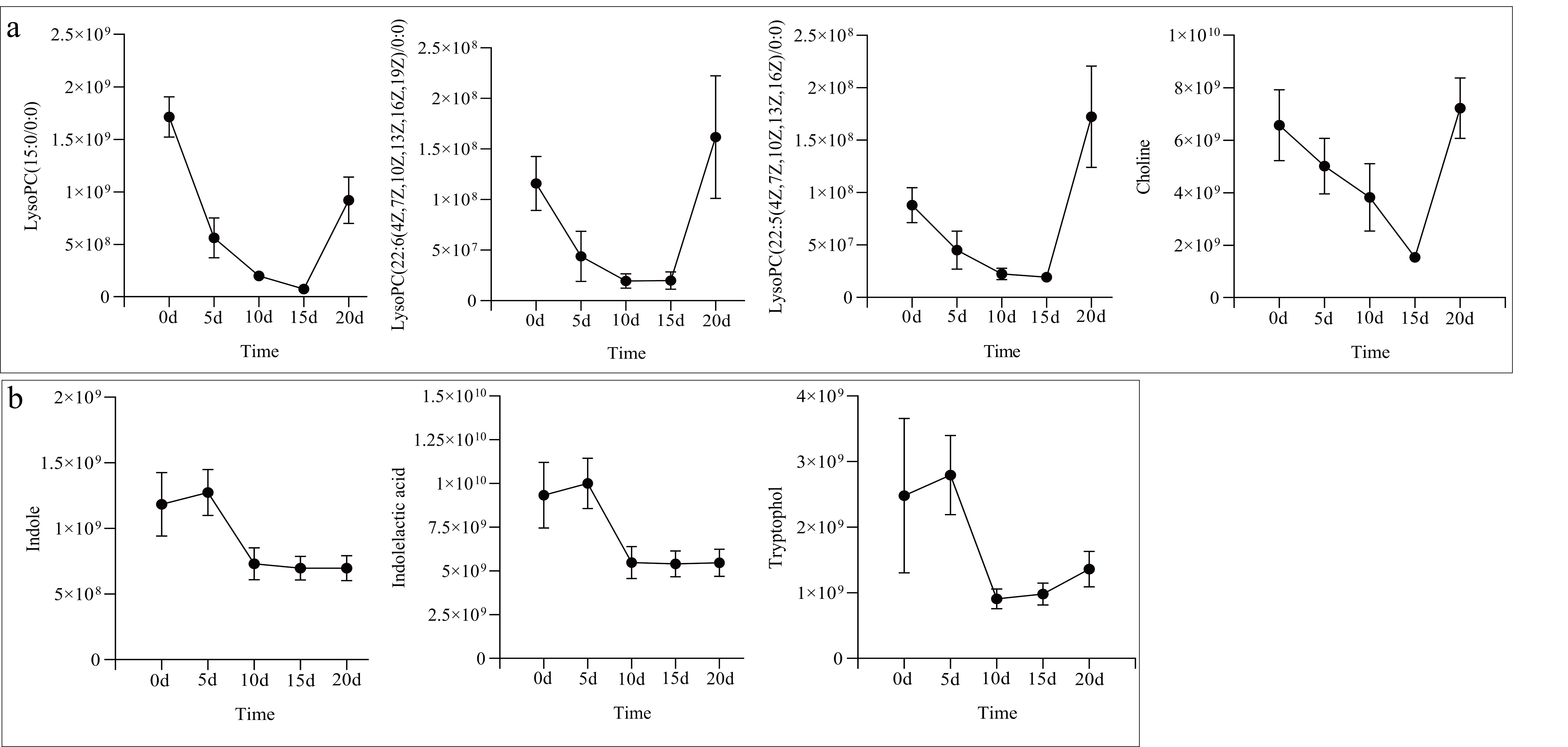


Fig S4. Differential metabolite dynamic changes. (a~b) Dynamics of differential metabolites in the positive ion mode during the occurrence of diarrhea over 20 days.





Fig S5. Differential metabolite dynamic changes. (a~e) Dynamics of differential metabolites in the negative ion mode during the occurrence of diarrhea over 20 days.

Table S1. Summary of Sample Numbers and Descriptions

| Number | Meaning | Time (d) | State | Sample Capacity |
| --- | --- | --- | --- | --- |
| NRM_0d | The samples were controlled normally throughout the process | 0 | Normal | 20 |
| NRM_5d |  | 5 | Normal | 20 |
| NRM_10d |  | 10 | Normal | 20 |
| NRM_15d |  | 15 | Normal | 20 |
| NRM_20d |  | 20 | Normal | 20 |
| CD_10d_0d | Diarrhea for 10 days | 0 | Normal | 20 |
| CD_10d_5d |  | 5 | Normal | 20 |
| CD_10d_10d |  | 10 | Diarrhea | 20 |
| CD_15d_0d | Diarrhea for 15 days | 0 | Normal | 20 |
| CD_15d_5d |  | 5 | Normal | 20 |
| CD_15d_10d |  | 10 | Normal | 20 |
| CD_15d_15d |  | 15 | Diarrhea | 20 |
| CD_20d_0d | Diarrhea for 20 days | 0 | Normal | 20 |
| CD_20d_5d |  | 5 | Normal | 20 |
| CD_20d_10d |  | 10 | Normal | 20 |
| CD_20d_15d |  | 15 | Normal | 20 |
| CD_20d_20d |  | 20 | Diarrhea | 20 |

Summary: During the sample collection process, the sample numbers are initially recorded using sequential Arabic numerals, and the condition of the calves is also noted. Once all the samples have been collected, the numbers are assigned based on the condition of the calves. The numbering rule is as follows: Samples starting with "NRM_" are from the control group that has never experienced diarrhea. Samples starting with "CD_" are from the experimental group that will experience diarrhea at a specific time point (e.g., 10 days). The grouping structure of "CD_10d_Group" follows the logic of grouping by the time of diarrhea occurrence, tracking the entire process from the beginning to the onset of diarrhea for each group. According to the diarrhea scoring standard (e.g., using a 1-4 point scale: 1 point for normal feces, 2 points for soft feces, 3 points for loose feces, and 4 points for watery feces), calves with a score of 3-4 were identified as diarrheic calves and included in the diarrhea group.

Table S2. Analysis Grouping Table

| Purpose of the analysis | Group Name | The sample numbers included | Sample Capacity | Group Description |
| --- | --- | --- | --- | --- |
| Analysis of Normal/Diarrheal States | NC_Group (Normal) | NRM_10d, NRM_15d, NRM_20d, CD_15d_10d, CD_20d_10d, CD_20d_15d | 120 | Samples in normal condition covering all time points |
|  | CD_Group (Diarrhea) | CD_10d_10d, CD_15d_15d, CD_20d_20d | 60 | Samples of diarrhea status covering all time points |
| Dynamic change | CD_10d_Group | CD_10d_0d(n=20),  CD_10d_5d(n=20),  CD_10d_10d(n=20), | 60 | The dynamic process of the group experienced diarrhea on the 10th day at different time points |
|  | CD_15d_Group | CD_15d_0d(n=20),  CD_15d_5d(n=20),  CD_15d_10d(n=20),  CD_15d_15d(n=20) | 80 | The dynamic process of the group experienced diarrhea on the 15th day at different time points |
|  | CD_20d_Group | CD_20d_0d(n=20),  CD_20d_5d(n=20),  CD_20d_10d(n=20),  CD_20d_15d(n=20),  CD_20d_20d(n=20) | 100 | The dynamic process of the group experienced diarrhea on the 20th day at different time points |

Summary: "Analysis of Normal/Diarrheal States" involves conducting data analysis between samples that experienced diarrhea within 10 d, 15 d, and 20 d and the normal samples. "Dynamic change" is determined by analyzing the dynamic changes that occur during diarrhea within 10 d, 15 d, and 20 d.

Table S3. Comparison of classification results of different models for normal and diarrhea microbiome data

| Index | ACC | Recall | F1-score | AUC |
| --- | --- | --- | --- | --- |
| LR | 0.778 | 0.708 | 0.68 | 0.76 |
| NN | 0.681 | 0.681 | 0.803 | 0.77 |
| RF | 0.806 | 0.917 | 0.863 | 0.88 |
| SLR | 0.764 | 0.292 | 0.452 | 0.84 |
| SVM | 0.806 | 0.875 | 0.857 | 0.85 |
| XGBoost | 0.847 | 0.849 | 0.891 | 0.91 |

Table S4. Comparison of differential metabolites showing upregulation and downregulation of positive and negative ions during 10 days of diarrhea occurrence

| Group | positive mode, up | positive mode, down | negative mode, up | negative mode, down |
| --- | --- | --- | --- | --- |
| CD_10d_0d vs  CD_10d_5d | 60 | 348 | 42 | 211 |
| CD_10d_0d vs CD_10d_10d | 218 | 178 | 133 | 94 |
| CD_10d_5d vs CD_10d_10d | 350 | 58 | 201 | 33 |

Table S5. Comparison of differential metabolites showing upregulation and downregulation of positive and negative ions during 15 days of diarrhea occurrence

| Group | positive mode, up | positive mode, down | negative mode, up | negative mode, down |
| --- | --- | --- | --- | --- |
| CD_15d_0d vs  CD_15d_5d | 68 | 410 | 32 | 282 |
| CD_15d_0d vs CD_15d_10d | 65 | 408 | 32 | 265 |
| CD_15d_0d vs CD_15d_15d | 350 | 58 | 175 | 59 |
| CD_15d_5d vs CD_15d_10d | 153 | 161 | 98 | 86 |
| CD_15d_5d vs CD_15d_15d | 428 | 56 | 290 | 14 |
| CD_15d_10d vs CD_15d_15d | 464 | 45 | 315 | 12 |

Table S6. Comparison of differential metabolites showing upregulation and downregulation of positive and negative ions during 20 days of diarrhea occurrence

| Group | positive mode, up | positive mode, down | negative mode, up | negative mode, down |
| --- | --- | --- | --- | --- |
| CD_20d_0d vs  CD_20d_5d | 50 | 355 | 41 | 218 |
| CD_20d_0d vs CD_20d_10d | 85 | 323 | 56 | 216 |
| CD_20d_0d vs CD_20d_15d | 58 | 379 | 28 | 246 |
| CD_20d_0d vs CD_20d_20d | 254 | 169 | 158 | 67 |
| CD_20d_5d vs CD_20d_10d | 154 | 82 | 71 | 51 |
| CD_20d_5d vs CD_20d_15d | 140 | 218 | 79 | 147 |
| CD_20d_5d vs CD_20d_20d | 389 | 39 | 243 | 15 |
| CD_20d_10d vs CD_20d_15d | 20 | 160 | 15 | 83 |
| CD_20d_10d vs CD_20d_20d | 370 | 36 | 249 | 9 |
| CD_20d_15d vs CD_20d_20d | 440 | 58 | 291 | 14 |
